# NF-*κ*B-Inducing Kinase Increases Renal Tubule Epithelial Inflammation Associated with Diabetes

**DOI:** 10.1155/2011/192564

**Published:** 2011-08-18

**Authors:** Yanhua Zhao, Srijita Banerjee, Wanda S. LeJeune, Sanjeev Choudhary, Ronald G. Tilton

**Affiliations:** ^1^Division of Endocrinology and Stark Diabetes Center, Department of Internal Medicine, The University of Texas Medical Branch, 301 University Baulevard, Galveston, TX 77555-1060, USA; ^2^Sealy Center for Molecular Medicine, The University of Texas Medical Branch, Galveston, TX 77555-1060, USA; ^3^Department of Ophthalmology & Visual Sciences, The University of Texas Medical Branch, Galveston, TX 77555-1060, USA

## Abstract

The impact of increased NF-**κ**B-inducing kinase (NIK), a key component of the NF-**κ**B activation pathways, on diabetes-induced renal inflammation remains unknown. We overexpressed NIK wild type (NIKwt) or kinase-dead dominant negative mutants (NIKdn) in HK-2 cells and demonstrated that RelB and p52, but not RelA, abundance and DNA binding increased in nuclei of NIKwt but not NIKdn overexpressed cells, and this corresponded with increases in multiple proinflammatory cytokines. Since TRAF3 negatively regulates NIK expression, we silenced TRAF3 by >50%; this increased nuclear levels of p52 and RelB, and transcript levels of proinflammatory cytokines and transcription factors. In HK-2 cells and mouse primary proximal tubule epithelial cells treated with methylglyoxal-modified albumin, multiple proinflammatory cytokines and NIK were increased in association with increased nuclear RelB and p52. These observations indicate that NIK regulates proinflammatory responses of renal proximal tubular epithelial cells via mechanisms involving TRAF3 and suggest a role for NF-**κ**B noncanonical pathway activation in modulating diabetes-induced inflammation in renal tubular epithelium.

## 1. Introduction

The concept that diabetes is a chronic inflammatory disease is supported by significant clinical and experimental data indicating that chronic, low-grade inflammation is a common denominator linking obesity, insulin resistance, atherosclerosis, dyslipidemia, and excessive glucose metabolism in diabetes [1–4]. Numerous diabetic stimuli, including elevated glucose, glycation products, and reactive oxygen species, have been shown to activate nuclear factor-*κ*B (NF-*κ*B) in relevant renal cells [5–7], contributing to the proinflammatory diabetic milieu. The NF-*κ*B family of transcription factors is controlled by distinct canonical and noncanonical activation pathways. The former plays an important role in the host innate immune response by inducing the expression of numerous proinflammatory cytokines, chemokines, adhesion molecules, growth factors, and inducible enzymes [8–11]. The latter plays an important role in the adaptive immune response, including secondary lymphoid organogenesis and lymphocyte maturation [12–14]. This pathway is mediated by NF-*κ*B-inducing kinase (NIK)·IKK*α* complexes that process p100 into p52 that translocates into the nucleus as a complex with RelB.

We and others have reported the increased expression of numerous NF-*κ*B-regulated proinflammatory/proangiogenic signaling cytokines, chemokines, and growth factors in the renal cortex of *db/db* mice, indicating a dysregulated and prolonged activation of the NF-*κ*B canonical pathway [15]. Additionally, we have observed several fold increases in NIK expression in the renal cortex of *db/db* mice that was localized to renal proximal tubular epithelial cells. The pathophysiological significance of this finding remains unknown, although NIK has been shown to be a potent proinflammatory kinase in a variety of cells and tissues. 

Based on our animal studies, we have suggested that diabetes activates both canonical and noncanonical NF-*κ*B pathways, leading to renal inflammation, and the diabetes-induced activation of the NF-*κ*B noncanonical pathway via the upstream NIK plays a causal role in prolonging the innate immune response activated by the canonical pathway. In this study, we have used human HK-2 cells and primary proximal epithelium (pPEC) harvested from mouse renal cortex to probe the role of noncanonical NF-*κ*B pathway activation in response to manipulation of NIK and TRAF3 levels as well as methylglyoxal-modified human serum albumin (MG-HSA), a stimulus that mimics the *in vivo* diabetic milieu.

## 2. Materials and Methods

### 2.1. Materials and Antibodies

All chemicals used were from Sigma. A list of primary and secondary antibodies used can be found in Tables 1 and 2.

### 2.2. Cell Culture

Human kidney cortex proximal tubular cells, HK-2 (ATCC), were grown and maintained in the recommended medium, Dulbecco's modified Eagle's medium/Ham's F12 (50/50) supplemented with 10% fetal bovine serum, L-glutamine, insulin, transferrin, sodium selenite, EGF (2.5 ng/mL), and pituitary extract (1.5 *μ*g/mL) in a humidified atmosphere of 5% CO_2_.

### 2.3. Primary Culture of Mouse Proximal Tubule Epithelial Cells (pPEC)

Renal cortical tubular epithelial cells were isolated and cultured using published techniques with modifications [16]. Briefly, 12–16-week C57BL/6 male mice were used to prepare primary renal proximal tubule cell cultures. Kidneys were sliced into coronal sections and cortical tissue separated from the medullary portion of each kidney under magnification with a dissecting microscope, minced, then washed 3x in ice cold DMEM-F12 media containing 0.1% BSA, followed by enzymatic digestion using 1% Worthington collagenase Type II and 0.25% soybean trypsin inhibitor. Cell suspensions were passed through 200 mesh followed by 325 mesh filters, then resuspended in 45% Percoll and centrifuged at ~27,000× g for 15 min at 4°C. Proximal tubule cells were sedimented to a layer immediately above the erythrocyte pellet. These cells were removed, centrifuged, washed to remove the remaining Percoll, then resuspended in Dulbecco's modified Eagle's medium/F-12 containing 50 units/mL of penicillin, 50 *μ*g/mL streptomycin, 10 ng/mL epidermal growth factor, 0.5 *μ*M hydrocortisone, 0.87 *μ*M bovine insulin, 50 *μ*M prostaglandin E_1_, 50 nM sodium selenite, 50 *μ*g/mL human transferrin, and 5 pM 3,3′,5-triiodo-L-thyronine. Cells were plated on Matrigel-coated coverslips, or plastic cell culture dishes coated with Matrigel, and maintained in an incubator at 37°C in 5% CO_2_. Cultures were left undisturbed for 48 hr, after which culture media was replaced every 2 days until cells achieved confluence. For all experiments, cells were used within three passages as described [16].

### 2.4. NIK Transfection

HK-2 cells were transfected with the eukaryotic expression vectors pCDNA-MycNIK or pCDNA-MycNIK dominant negative, encoding NIK Thr^559^Phe site mutant as described previously [17], or empty vector plasmid DNA (pCDNA) using either lipofectamine and Plus Reagent (Invitrogen, Carlsbad, CA) or by electroporation using Cell Line Nucleofector Kit V (Amaxa Biosystems) according to the manufacturers' instructions. Cells were cultured for variable times as indicated below, and either whole cell lysates or cytosolic and nuclear extracts were prepared.

### 2.5. Gene Silence by siRNA

TRAF3 siRNA and nontargeting control siRNA (Dharmacon Smart Pools, Lafayette, CO) (SMART pools) were transfected into HK-2 cells by TransIT-siQUEST Transfection Reagent (Mirus Corp., Madison, WI) at 50 nmol/L or 100 nmol/L final concentration according to the manufacturer's instructions. Up to 72 hr posttransfection, cells were used for measuring TRAF3 knockdown and cytokine production.

### 2.6. Preparation of Methylglyoxal-Modified Serum Albumin

Human serum albumin (HSA) minimally modified by methylglyoxal (MG) was prepared as described (3) by incubation of the protein (100 *μ*M) in sodium phosphate buffer (100 mM, pH 7.4 and 37°C) with 500 *μ*M methylglyoxal for 24 hours. MG-modified protein was dialyzed against ammonium bicarbonate buffer (30 mM, pH 7.9 and 4°C) for 24 hrs, followed by a PBS dialysis buffer for another 24 hrs; sterilized by filtration (0.22 *μ*m), aliquoted, and stored −80°C until used. The extent of methylglyoxal modification was determined using a Hitachi L-8800 amino acid analyzer. Unmodified protein was processed similarly for control experiments.

### 2.7. Preparation of Cell Lysates

For whole cell lysates, culture media was removed, plates rinsed with ice cold PBS, and cells scraped using ice cold PBS (1 mL/100 mm dish) and transferred to eppendorf tubes, then spun at 2000 rpm for 2 min at 4°C. The supernatant was removed and 100–500 *μ*L 1x RIPA buffer-containing protease (Sigma, catalog #P8340) and phosphatase (1 mM orthovanadate and 30 mM sodium fluoride) inhibitors was added to cell pellet for 30 min on ice with periodic vortexing, followed by centrifugation for 15 min at 4°C at 13,200 rpm. The supernatant was stored as whole cell lysate at −80°C. 

For cytosolic and nuclear extracts, the cell pellet was obtained from culture plates as above, and 100–300 *μ*L solution A (50 mM HEPES, pH 7.9, 10 mM KCl, 1 mM EDTA, 1 mM EGTA, protease inhibitor cocktail (Sigma), phosphatase inhibitors [1 mM orthovanadate and 30 mM sodium fluoride], 1 mM DTT, and 0.5 mM phenylmethylsulphonylfluoride [PMSF]) was added with gentle mixing by pipeting, then incubated on ice for 10 min. 10% NP-40 was added for a final concentration of 0.5%, and the cells immediately spun for 7 min at 1,000 g at 4°C. The supernatant was used as cytosolic extract. The pellet was gently resuspended in 1 : 1 (v : v) solution B (50 mmol/L HEPES, pH 7.9, 10 mM KCl, 1 mM EDTA, 1 mM EGTA, 34% sucrose, protease and phosphatase inhibitors, 1 mM DTT, and 0.5 mM PMSF), then spun at 7400 g for 5 min at 4°C. The supernatant was removed and discarded, and the pellet was resuspended in 10 *μ*L solution A followed by 40 *μ*L of solution C (50 mM HEPES, pH 7.9, 500 mM KCl, 1 mM EDTA, 1 mM EGTA, 10% glycerol, protease and phosphatase inhibitors, 1 mM DTT, and 0.5 mM PMSF) with gentle vortexing. The suspension was allowed to sit on ice for 30 min, vortexing every 5 min, then spun at 16, 100 g for 20 min at 4°C. The supernatant was used as nuclear extract. Protein concentrations of nuclear and cytosolic extracts were determined using the Bio-Rad (Hercules, CA) protein assay with bovine albumin standards, and both samples were assayed for cytosolic (*β*-tubulin) and nuclear (lamin B) proteins.

### 2.8. Western Immunoblot Analysis

Whole cell, nuclear and cytoplasmic lysates were electrophoresed on 7.5 or 10% SDS-polyacrylamide gels and analyzed by immunoblotting after transfer to nitrocellulose membranes (Bio-Rad Laboratories, Hercules, CA), using primary antibodies (Table 1) according to each manufacturer's instructions, followed by species-specific secondary antibodies tagged with a fluorescent dye (IR Dye 800; Rockland) at a 1 : 5,000 dilution (Table 2). Densitometric quantitation of each protein was performed using the LI-COR Bioscience Odyssey Imaging System with infrared fluorescence detection or with an ECL system onto film. *β*-actin was measured on every blot (using an IR Dye 700 with the LI-COR system) for evaluation of protein loading.

### 2.9. DNA Binding Assay

TransAM NF-*κ*B Family Transcription Factor Assay Kit (Cat no. 43296, ActiveMotif) was used for the DNA-binding assay according to the manufacturer's instructions. Briefly, oligonucleotide containing an NF-*κ*B consensus binding site was immobilized on a 96-well plate, and activated NF-*κ*B subunits contained in nuclear extract were added and specifically bound to this oligonucleotide. Antibodies directed against specific NF-*κ*B subunits (RelA, RelB, p50, p52) complexed with the oligonucleotide were added, followed by rinsing and addition of a secondary antibody conjugated to horseradish peroxidase for spectrophotometric quantification.

### 2.10. Quantitative Real-Time PCR (qRT-PCR)

Total RNA was extracted using acid guanidinium-phenol extraction (TRI reagent; Sigma, T9494) according to the manufacturer's instructions. Two to five micrograms of RNA were reverse transcribed using SuperScript III (Invitrogen Corp.) in a 20-*μ*L reaction mixture. One microliter of cDNA product was diluted 1 : 2, and 2 *μ*L were amplified in a 20-*μ*L reaction mixture containing 12.5 *μ*L of SYBR green supermix (Bio-Rad) and 0.2 *μ*M each of forward and reverse gene-specific primers (SuperArray Bioscience Corp), aliquoted into 96-well, 0.2-mm thin-wall PCR plates. Plates were denatured for 90 sec at 95°C, then subjected to 40 cycles of 15 sec at 94°C, 60 sec at 60°C, and 1 min at 72°C in an iCycler (Bio-Rad). For each experimental sample, triplicate reactions were conducted in 96-well plates. The ΔΔCt method was used to analyze the experimental data with GAPDH or *β*-actin used as internal standard.

### 2.11. Cytokine Measurements

Multiple cytokines and chemokines were measured on aliquots of tissue culture supernatant or cytosolic extracts using customized human or mouse cytokine/chemokine kits (Millipore) with the Bio-Plex system, used with the Luminex xMAP technology according to the manufacturer's instructions (Bio-Rad Laboratories, Hercules, CA). Eight-point standard curves were performed for each cytokine using the same Luminex bead technology.

### 2.12. Statistical Analysis

All results are expressed as means ± SD or SEM as indicated. For assessment of cytokine data, one-way and two-way ANOVA tests were performed to evaluate overall group differences. This was followed by appropriate post hoc tests to determine pairwise significance. For all other two sample comparisons, a Student's *t*-test was performed after checking for variance distribution via Levene's test. In all cases, *P* < 0.05 was considered significant.

## 3. Results

### 3.1. MG-HSA Increases NF-*κ*B-Mediated Cytokine Production in HK-2 Cells and Mouse pPEC in Tissue Culture

**Figure 1:**
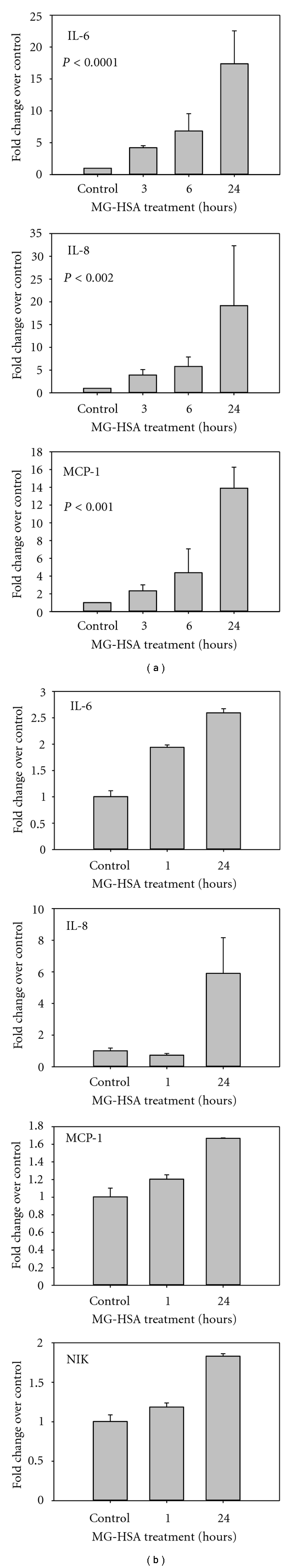
*MG-HSA increased NF-*κ*B-mediated cytokine production in HK-2 and primary proximal tubular epithelial cells: *(a) Cytokines were measured on aliquots of tissue culture supernatant using customized human multiplex kits, using the Bio-Plex system according to the manufacturer's instructions. Experiments were performed twice in triplicate, and data normalized to control (HK-2 cells exposed to unmodified HSA) values. Data represent mean ± SD of two independent experiments and compared using 1-way ANOVA (significance values are shown within each panel). (b) Renal primary tubular epithelial cells were treated with MG-HSA (5 *μ*M) for various times, and expression levels of IL-6, IL-8, MCP-1, and NIK were measured in a single experiment performed in triplicate (error bars represent SD of intra-assay variability) by using Q-RT-PCR. Data were normalized to GAPDH and expressed as fold change as compared to the untreated cells (Control).

HK-2 cells were grown to 80% confluence then treated with 5 *μ*M MG-HSA for up to 24 hours in tissue culture. Figure 1(a) demonstrates time-dependent, increased production of NF-*κ*B-related cytokines, including IL-6, IL-8, and MCP-1, measured in aliquots of the tissue culture media. Similar results were obtained using RT-PCR assay for cytokines in MG-HSA-treated primary proximal tubules of mouse kidney cortex exposed to the same concentration of MG-HSA, including increased production of NIK, measured over a 24-hour time period (Figure 1(b)).

### 3.2. NIK Overexpression in HK-2 Cells Increases Noncanonical NF-*κ*B Pathway Activation

**Figure 2:**
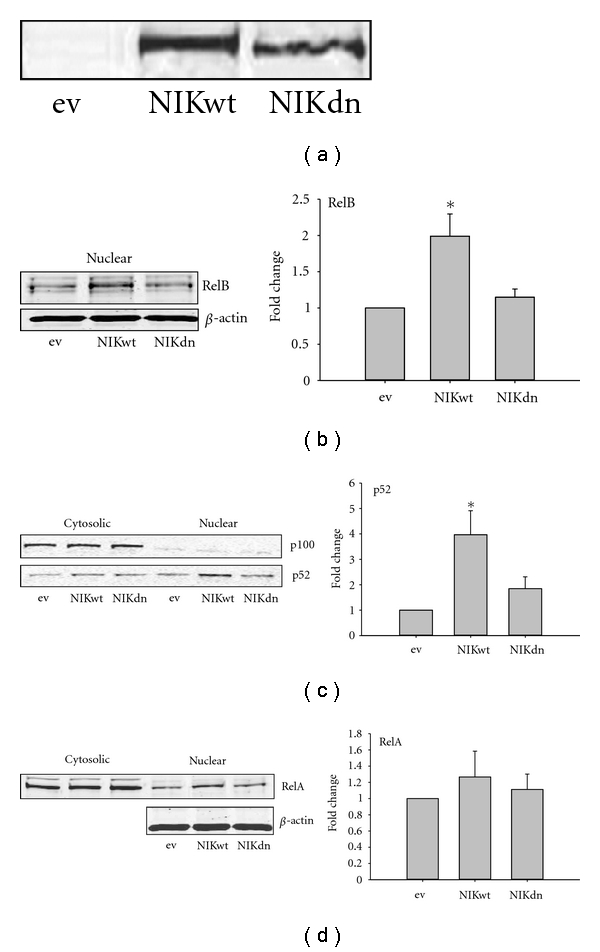
*NIK overexpression in HK-2 cells increases noncanonical NF-*κ*B pathway activation**: ***HK-2 cells were transfected with the eukaryotic expression vectors pCDNA-MycNIK (NIKwt) or pCDNA-MycNIK dominant negative (NIKdn), encoding NIK Thr^559^Phe site mutant or empty vector (ev) plasmid DNA (pCDNA). (a) Representative Western blot showing expression of NIKwt and NIKdn transfected at the same plasmid concentration; (b) Western blot showing nuclear RelB on the left and quantification of 4 separate experiments on the right; (c) Representative Western blot showing cytosolic and nuclear p100 and p52 on the left and quantification of nuclear p52 from 5 separate experiments on the right. P100 is predominantly cytosolic and despite the increase in nuclear p52, it did not change in abundance. (d) Representative Western blot showing cytosolic and nuclear RelA on the left and quantification of nuclear RelA from 4 separate experiments on the right. Bar graph data are presented as mean ± SE; Student's *t*-test versus empty vector control (ev): *P < 0.05.

**Figure 3:**
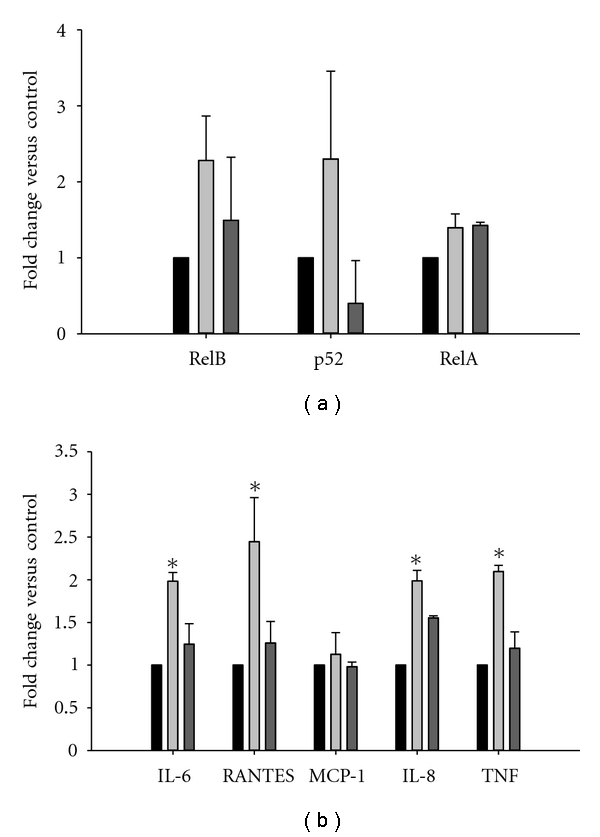
*DNA-binding assay and cytokine measurements:* (a) TransAM NF-*κ*B Family Transcription Factor Assay was used to measure RelA, RelB, and p52 DNA binding following NIKwt and NIKdn overexpression in HK-2 cells. For each Rel protein, ev (left), NIKwt (middle), and NIKdn (right) are shown. Data represent two separate experiments. One-way ANOVA: *P* < 0.02. (b) Cytokine measurements determined using tissue culture media obtained from the same experiments. Data represent two separate experiments. Two-way ANOVA: *P* < 0.008. Student's *t*-test versus empty vector control: *P < 0.05.

**Figure 4:**
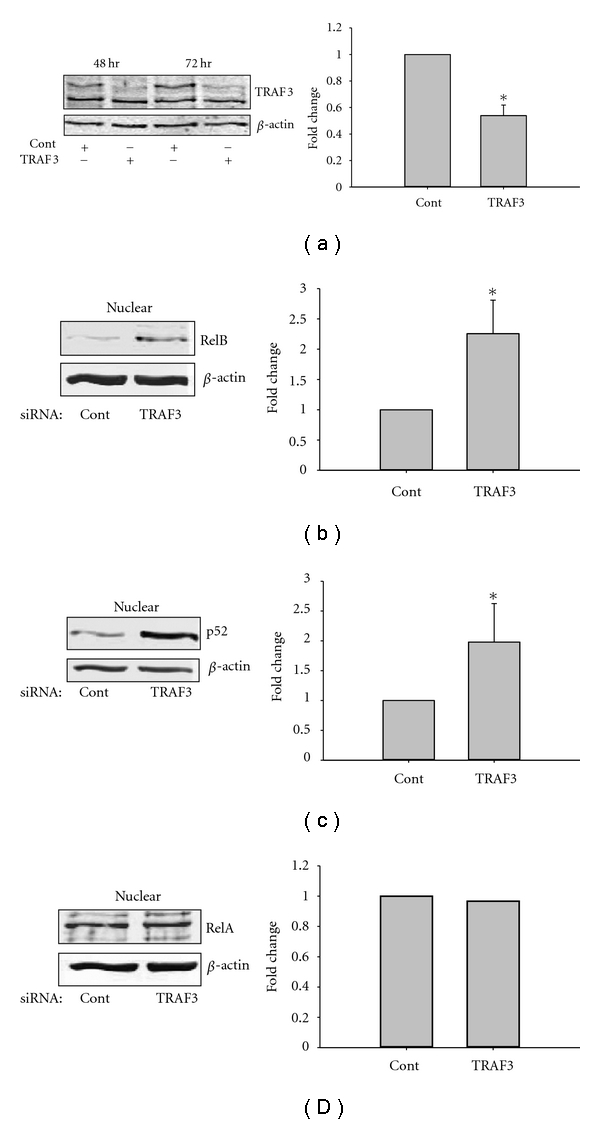
*TRAF3 siRNA knockdown: *TRAF3 was knocked down using specific siRNA by TransIT-siQUEST Transfection Reagent in HK-2 cells. (a) Representative Western blot demonstrating effects of 100 nM TRAF3 siRNA after 48 and 72 hours with *β*-actin as a loading control is shown on the left and quantification of the pooled 48 and 72 hour data is shown on the right. All subsequent experiments used 100 nM siRNA. (b) Representative Western blot showing nuclear RelB on the left and quantification of 2 separate experiments on the right; (c) Representative Western blot showing nuclear p52 on the left and quantification of 3 separate experiments on the right; (d) Representative Western blot showing nuclear RelA on the left and quantification of one experiment on the right. Student's *t*-test versus empty vector control: *P < 0.05.

Overexpression of NIKwt and NIKdn plasmids in HK-2 cells is shown in Figure 2(a). The increased NIKwt protein was associated with 2-fold increases in nuclear RelB (Figure 2(b)) and 4-fold increases in nuclear p52 (Figure 2(c)). P100 was predominantly expressed in the cytoplasm, and despite the significant increase in p100 processing to p52, it did not change in abundance. Insignificant increases in nuclear Rel A were observed with no change in cytosolic expression levels (Figure 2(d)). Overexpression of NIKdn did not increase RelA, RelB, or p52 nuclear protein. The increased nuclear protein was associated with >2-fold increases in RelB and p52 DNA binding but no increase in RelA DNA binding (Figure 3(a)). Most cytokine levels in NIKwt-overexpressed cells were increased 2-fold, while NIKdn expression had no effect (Figure 3(b)). In contrast to the effects of MG-HSA (shown in Figure 1), overexpression of NIKwt did not increase MCP-1 production.

### 3.3. TRAF3 siRNA Knockdown

Low basal levels of NIK are maintained in cells by regulating its turnover by TRAFs. In nonstimulated cells, TRAF-3 recruits NIK to the complex containing TRAF2 and cIAP1/2. In this complex, NIK undergoes ubiquitination by cIAP1/2 resulting in rapid proteosomal degradation. However, in the presence of noncanonical stimuli, cIAP1/2 ubiquitinates and promotes degradation of TRAF3, thereby releasing NIK from negative regulation by TRAF and leading to its stabilization and accumulation in the cells. To understand the physiological relevance of TRAF3-mediated regulation of NIK steady-state levels, TRAF3 was knocked down using specific siRNA by TransIT-siQUEST Transfection Reagent as per the manufacturer protocol. TRAF3 knockdown was effective at 100 nM plasmid concentration after 48- and 72-hours shown by Western blot in Figure 4(a). An average of the 48 and 72 hour western blots obtained from two separate experiments is tabulated on the right in Figure 4(a). 

**Figure 5:**
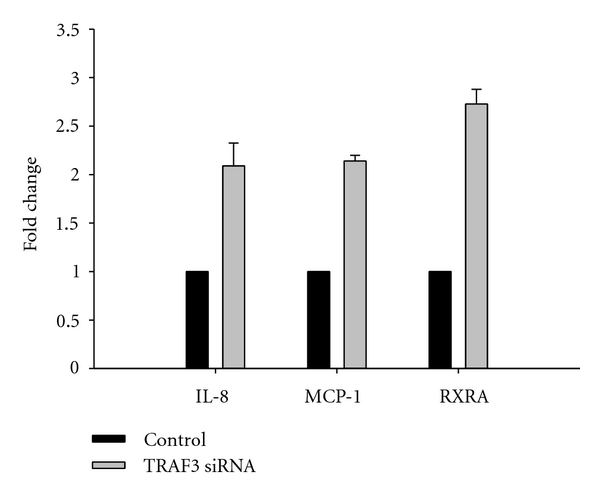
*TRAF3 silencing increases proinflammatory proteins: *TRAF3 was knocked down using specific siRNA in HK-2 cells, and cytokines (IL-8 and MCP-1) and retinoid x receptor alpha (RXRA) were measured by RT-PCR in a single experiment performed in triplicate (error bars represent SD of intra-assay variability).

TRAF3 silencing increased nuclear content of RelB (Figure 4(b)) and p52 (Figure 4(c)) without affecting nuclear RelA (Figure 4(d)). Cytokine levels in HK-2 measured by RT-PCR, including IL-8 and MCP-1, as well as the retinoid acid receptor alpha (RXRA) were increased 2- to 2.5-fold by TRAF3 silencing (Figure 5).

## 4. Discussion

NF-*κ*B is a family of highly inducible cytoplasmic DNA binding proteins that includes the transactivating subunits, RelA, RelB, c-Rel, and the posttranslationally processed DNA-binding subunits, NF-*κ*B 1 (p50) and NF-*κ*B2 (p52) [18]. NF-*κ*B dimers remain sequestered in the cytoplasm by interacting with a group of inhibitory ankyrin repeat-containing proteins, collectively referred to as I*κ*Bs (I*κ*B*α*, I*κ*B*β*, I*κ*B*γ*, p100, and p105) [19]. Recently, it has been appreciated that NF-*κ*B activation can be controlled by at least two separate and independent pathways: the “canonical pathway” mediated by the I*κ*B kinase (IKK, a complex of two catalytic subunits, IKK*α* and IKK*β* and the regulatory subunit, IKK*γ*) [20] and the “noncanonical pathway” mediated by a complex of IKK*α* and NIK. Stimuli activating NF-*κ*B via the canonical pathway activate IKK kinase, resulting in I*κ*B*α* phosphorylation at specific N-terminal serine residues targeting them for proteasomal degradation [21]. This process releases sequestered RelA·p50 to enter the nucleus.

To date, the noncanonical pathway has been shown to play a role in the adaptive immune response, including secondary lymphoid organogenesis, the induction of genes involved in this process, and lymphocyte maturation [12–14]. Recently, it has been observed that the noncanonical pathway can be activated in response to specific stimuli, including lymphotoxin *β* [LT*β*] [22, 23], CD40 ligand [24], DNA virus infection [25], and B-cell activating factor [BAFF] [12, 26, 27]. Interestingly, neither IKK*γ* nor IKK*β*, key regulators of the canonical pathway, are required for activation of the noncanonical pathway [12, 23]. Rather, a kinase complex consisting of NIK and IKK*α* activates posttranslational processing of p100 into the 52 kDa-active DNA-binding isoform. Newly formed p52 dimerizes with cytoplasmic RelB and translocates into the nucleus. In this pathway, NIK serves to activate IKK*α* and provides a docking site to recruit both p100 and IKK*α* [23]. NIK therefore is an essential component of the noncanonical NF-*κ*B activation pathway. 

NIK is a 110 kDa ser-thr kinase first identified as a TRAF-binding protein in yeast two hybrid screening. Because of its ability to activate both IKK*α* and IKK*β*, NIK has been extensively studied as an upstream kinase regulating NF-*κ*B activation pathways [22, 23, 28–30]. The observation that an NIK·IKK*α* complex is responsible for proteolytic processing of p100 to generate p52 has generated significant interest in understanding the molecular role of NIK as a regulator of NF-*κ*B activation. However, the physiological and pathophysiological role of NIK remains mostly unexplored, and very few *in vivo *studies have explored the impact of increased NIK activation. It has been reported in pig models of ischemia-reperfusion injury and in delayed graft function in patients receiving kidney transplants [31]. In the latter, activation of NIK occurred within proximal tubular epithelial cells, in a pattern strikingly similar to the diabetes-induced changes we have reported. NIK activity also can be induced by thrombin in cultured proximal tubular epithelial cells [31].

We have postulated that diabetes activates both canonical and noncanonical NF-*κ*B pathways, leading to renal inflammation. Since NF-*κ*B activation is well known to be rapidly arrested, an important, unanswered question is why this does not occur in diabetic tissues, where canonical pathway activation of the innate immune response persists chronically, contributing significantly to the inflammatory/angiogenic phenotype reported in diabetic tissues. Since this is very difficult to test *in vivo*, and while NIK is constitutively produced, but is kept at very low intracellular levels by TRAF3, we utilized overexpression systems in tissue culture to probe the impact of elevated NIK levels on canonical versus noncanonical inflammation. We have shown that a relevant diabetic stimulus, methylglyoxal-modified human serum albumin, can activate NF-*κ*B to increase cytokine production, and that this is associated with increases in NIK levels. Furthermore, increasing endogenous NIK levels via transfection of full-length NIK sufficient to overwhelm endogenous TRAF3-mediated regulatory mechanisms, or silencing TRAF3, both lead to increased cytokine production similar to that observed with relevant diabetic stimuli. Importantly, this was associated with increases in the amount as well as increases in DNA binding of nuclear NF-*κ*B proteins related to the noncanonical activation pathway (RelB and p52) without measurable changes in nuclear RelA. These changes were not linked to the plasmid transfection, since overexpression of NIK protein lacking kinase activity did not result in similar RelB and p52 nuclear changes.

## 5. Conclusions

Elevating cellular levels of NIK by overexpression or by silencing TRAF3, an endogenous posttranslational regulatory mechanism for NIK, resulted in increased nuclear translocation of RelB and p52, and increased DNA binding of these Rel proteins relative to the canonical RelA protein. This resulted in increased cytokine production similar to that observed with methylglyoxal-modified albumin. These observations indicate that NIK regulates proinflammatory responses of renal proximal tubular epithelial cells via mechanisms involving TRAF3 and suggest a role for NF-*κ*B noncanonical pathway activation in modulating diabetes-induced inflammation in renal tubular epithelium.

## Figures and Tables

**Table 1:** List of antibodies used against the protein targets.

Target	Species	Dilution	Company	MW (kDa)
NIK	Rabbit	1 : 1,000	Cell signaling 4994	>100
TRAF3	Rabbit	1 : 500	Santa Cruz sc949	65
p52/p100	Mouse	1 : 1,000	Upstate 05-361	52/100
RelB	Rabbit	1 : 500	Santa Cruz sc226	68
RelA	Rabbit	1 : 2,000	Rockland100-4165	65
*β*-tubulin	Rabbit	1 : 5,000	Santa Cruz sc9104	55
Lamin B	Goat	1 : 5,000	Santa Cruz sc6216	67
*β*-actin	Mouse	1 : 20,000	Sigma A1978	42
*β*-actin	Rabbit	1 : 20,000	Sigma A2103	42

**Table 2:** Secondary antibodies.

Name of the secondary antibody	Company
IRDye 800 Affinity Purified Antimouse IgG (goat)	Rockland, 610-132-121
IRDye 800 Affinity Purified Antigoat IgG (donkey)	Rockland, 605-731-125
IRDye 800 Affinity Purified Antirabbit IgG (goat)	Rockland, 611-132-122
Alexa Fluor 680 goat antimouse IgG	Invitrogen A21057
Alexa Fluor 680 donkey antigoat IgG	Invitrogen A21084
Alexa Fluor 680 goat antirabbit IgG	Invitrogen A21109

All secondary antibodies were used at a 1 : 5,000 dilution.

## Abbreviations

DTT: Dithiothreitol
I*κ*B: Inhibitor of *κ*B
IKK: Inhibitor of *κ*B kinase
NF-*κ*B: Nuclear factor-*κ*B
NIK: NF-*κ*B-inducing kinase
pPEC: (mouse) Primary proximal tubule epithelial cell
PMSF: Phenylmethylsulphonylfluoride.
